# Preventive measures against the COVID-19 pandemic in Mexico: A cross-sectional study

**DOI:** 10.3389/fpubh.2022.932010

**Published:** 2022-10-11

**Authors:** Juan Carlos Ibarrola-Peña, Francisco José Barbosa-Camacho, Yolanda Lorelei Almanza-Mena, Jonathan Matías Chejfec-Ciociano, Emilio Alberto Reyes-Elizalde, Olaya Moramay Romero-Limón, Rafael Zaragoza-Organista, Enrique Cervantes-Pérez, José Héctor Sapién-Fernández, José Aldo Guzmán-Barba, Paola Flores-Becerril, Itzel Ochoa-Rodríguez, Rodrigo Nájar-Hinojosa, Andrea Estefania Cueto-Valadez, Tania Abigail Cueto-Valadez, Martín López-Zendejas, Clotilde Fuentes-Orozco, Gabino Cervantes-Guevara, Roberto Carlos Miranda-Ackerman, Alejandro González-Ojeda

**Affiliations:** ^1^Unidad de Investigación Biomédica 02, Hospital de Especialidades del Centro Médico Nacional de Occidente, Instituto Mexicano del Seguro Social, Guadalajara, Jalisco, Mexico; ^2^Centro Universitario de Tonalá, Universidad de Guadalajara, Guadalajara, Mexico; ^3^Departamento de Nutrición Clínica, Instituto Nacional de Ciencias Médicas y Nutrición “Salvador Zubirán”, Ciudad de Mexico, Mexico; ^4^Department of Internal Medicine, Hospital San Javier, Guadalajara, Mexico; ^5^Departamento de Bienestar y Desarrollo Sustentable, Centro Universitario del Norte, Universidad de Guadalajara, Guadalajara, Jalisco, Mexico; ^6^Department of Gastroenterology, Hospital Civil de Guadalajara “Fray Antonio Alcalde”, Universidad de Guadalajara, Guadalajara, Mexico

**Keywords:** health behavior, pandemic, risk-taking, Mexico, COVID-19

## Abstract

**Introduction:**

Understanding how Mexicans behave during the pandemic could present a complete picture of the phenomenon in our country and provide better management of it.

**Objective:**

This study aimed to analyze the Mexican population's behavior and preventive measures.

**Methods:**

This was a cross-sectional study in which a total of 4,004 participants from the general population responded to the survey.

**Results:**

Almost 99% of the participants mentioned knowing the symptoms of COVID-19. Although 77.5% of participants considered that they followed proper social distancing measures, 60% of them mentioned that they knew at least six individuals who did not follow social distancing measures. Furthermore, 96.2% of participants reported using preventive measures at least 50% of the time. Only 51.3% used a certified mask.

**Conclusion:**

The COVID-19 pandemic outcomes in Mexico are the result of multiple negative factors, such as high rates of comorbidities, high number of people living together at home, many people breaking social isolation, and most of the population using non-certified preventive measures that may not be effective enough.

## Introduction

Atypical pneumonia cases of unknown origin were reported in Asia at the end of 2019. A new coronavirus was identified as the etiologic agent (1). The WHO reported a new viral pneumonia caused by SARS-CoV-2 in December 2019. The virus wreaked havoc first in Asia, then spread to the rest of the world, hitting Latin America in late February 2020, and is now spreading to over 42 million rural indigenous people (2).

Studying the behavior of the Mexican population during previous pandemics (Influenza A H1N1 pandemic), the Mexican government carried out preventive measures on time such as closing schools, mandating the use of face masks, and encouraging the general population to avoid social contact (3). Mexico was reported as one of the countries with the highest compliance with preventive measures; nearly 70% of participants used face masks as a preventive measure in addition to adopting social distancing behavior (4).

This time, however, the Mexican government's epidemiological strategies were inconsistent and ineffective. The first COVID-19-infected patient in Mexico was announced in February 2020 (5). In March, the Mexican Secretariat of Health predicted 2,50,656 COVID-19 cases, which is roughly 0.19% of Mexico's total population of 130.8 million. The Mexican government's official website reported 23,58,167 cases by March 2021 (6). Despite the WHO's recommendations (7), the president repeatedly invited its population to break social isolation (8). Furthermore, due to the high prevalence of comorbidities and deficiencies in public healthcare systems, Mexicans, like most Latinos, are particularly vulnerable to rapid virus spread. Furthermore, Mexico reported the highest fatality rate in Latin America at 8.5%, followed by Peru at 3.5% (9).

This study aimed to identify the risk factors favoring the COVID-19 contagion by studying the Mexican population's quarantine behavior and preventive measures. As a secondary aim, we sought to identify areas that needed reinforcement to develop strategies for a successful plan for upcoming pandemics.

## Methods

### Study design

This was a cross-sectional survey study where we inquired about the general Mexican population's behavior during the quarantine, such as using preventive measures (face masks, alcohol-based hand sanitizers, and face shields).

### Study sample

A total of 4,029 participants were surveyed, but only 4,004 were included in this study. The study population was selected for the convenience of using digital media. We used a Snowball recruiting method, where the general population was asked to share the survey with their friends and family. The inclusion criteria were Mexican citizens or residents of Mexico and >15 years of age. The exclusion criteria were < 15 years of age, those with incomplete surveys, tourists currently staying in the country, or Mexican but residing in another country.

### Sample size

According to Zazueta et al. (10), during the COVID-19 pandemic, face mask usage was reported to be up to 89%. According to Bults et al. (4), the face mask usage during the H1N1 pandemic was of 71%. The sample size was calculated using the Kelsey formula: $\frac{{\left(Z\alpha +Z\beta \right)}^{2}*p\left(1-p\right)\left(r+1\right)}{r{\left(p0-p1\right)}^{2}}$ obtaining a sample size of 124 minimum participants.

### Survey instrument

The survey instrument comprised a questionnaire that included demographic data, such as sex, age, school education level, and occupation. The survey also included questions about illnesses; consumption of substances such as alcohol, tobacco, coffee, and drugs; housing conditions; daily coexistence with people; use of preventive measures against COVID-19; confidence in the Mexican health sector; and general knowledge of the disease. The survey was completed anonymously and voluntarily using Google Forms. The survey included demographical variables such as sex, age, educational level, occupation, residency state, comorbidities, number of comorbidities, substance use, and the number of substance types used. The *Knowledge of COVID-19* section included the question: “Do you stay informed about the pandemic in your country and state?”. The *Daily coexistence with people* section included the questions: “How many people live in your household?” and “How often do they find themselves in need to break social isolation and leave home?”. The *Breaking social isolation* included the questions “Do you consider that you are following the proper rules for social distancing?”, “How many people in your closest circle do you consider not to be following adequate social distancing measures and behaving irresponsibly?”, and “Based on your daily activities, what is the risk that you think you have of contracting COVID-19?”. The *Personal preventive measures against COVID-19* included the questions “How often do you use preventive measures against COVID-19?” (face masks, eyewear, and alcohol-based sanitizer) and “Do you use certified preventive measures?” (certified or not certified). The *COVID-19 screening* section included the questions “Have you undergone COVID-19 testing?” and “Do you know anybody who has presented symptoms of or has been diagnosed?”. The *Mexican health-care system* section: included the question “Do you believe that the private and public hospitals in Mexico are prepared to face the pandemic crisis?”. The survey can be found in Additional file 1.

### Data analysis

The data were analyzed using IBM SPSS Statistics (version 23.0 for Windows). Proportions, means, and standard deviations were included for the descriptive analyses. The inferential analysis of categorical variables was performed using the chi-square test, Fisher's exact probability test, or variance analysis as appropriate. The student's *t*-test was used to analyze continuous variables. A probability level of *p* < 0.05 was considered significant.

## Results

A total of 4,029 participants responded to the survey. Twenty-five surveys were excluded from the study due to being incomplete, leading to 4,004 participants: 2,669 female (66.7%) and 1,335 male (33.3%) participants. The mean participant age was 30.01 ± 13.72 years. The participants were divided into two levels of education groups: an elementary education group (EEG), which included 987 participants (24.6%) with primary and high school level education, and a higher education group (HEG), which included 3,017 participants (75.3%) with college and university level education. The complete demographic characteristics of the participants are presented in Table 1.

**Table 1:** Participant demographic characteristics.

	**Sample (*n* = 4,004)**
**Sex**, ***n*** **(%)**
Male	1,335 (33.3)
Female	2,669 (66.7)
**Age, years**
Mean (standard deviation)	30.01 (13.72)
18–25	1,238 (30.9)
26–50	2,037 (50.9)
50 or more	729 (18.2)
**School education**
Elementary	987 (24.7)
Higher	3,017 (75.3)
**Occupation**
Formal job	2,351 (58)
Informal job	302 (7.5)
Student	822 (20.5)
Retired	135 (3.4)
Neither working nor studying	150 (3.7)
Other	244 (6.1)
**Residency state**
Jalisco	2,240 (55.94)
Other	1,764 (44.05)
**Comorbidities**
None	2,247 (56.11)
Overweight	1,272 (31.76)
Diabetes	165 (4.12)
Hypertension	330 (8.24)
Other	482 (12.03)
**Number of comorbidities**
None	2,247 (56.11)
1	1,153 (28.79)
2 or more	650 (16.23)
**Substance use**
None	1,119 (27.94)
Tobacco	538 (13.43)
Alcohol	1,129 (28.19)
Cannabis or other drugs	123 (3.07)
Coffee	2,199 (54.92)
**Number of substance types**
1	1,961 (48.97)
2	715 (17.85)
3	209 (5.21)

Preventive measures against the COVID-19 pandemic in Mexico: A cross-sectional study, México, 2020–2021.

### Knowledge of COVID-19

For the question “Do you stay informed about the pandemic in your country and state?” only 2,392 participants (59.7%) answered “yes,” with a significant difference between the number of such respondents in the EEG and HEG (*p* < 0.001) [510 participants (51.6%) and 1,882 participants (62.3%), respectively]. A total of 3,954 participants (almost 99%) mentioned knowing the symptoms of COVID-19, and almost 90% of the participants mentioned knowing where they could go in case of having current symptoms of COVID-19.

### Daily co-existence with people

In response to the question “How many people live in your household?”, 2,728 participants (68.1%) mentioned 3–5 co-habitants and nearly 3,091 participants (77.1%) lived with >3 co-habitants. The tendency to live with a greater number of people (>3 co-habitants) was more noticeable in the EEG than in the HEG [828 participants (83.8%) vs. 2,263 participants (75%), respectively]. Moreover, a similar trend was observed for “>6 co-habitants” [124 participants (12.5%) in the EEG vs. 239 participants (7.9%) in the HEG]. When asked “How often do they find themselves in need to break social isolation and leave home?”, we divided the sample into two groups: 3,502 participants (87.4%) stated that at least one household member must break the isolation per week [2,774 participants (69.3%) with “1–2 co-habitants” and 728 participants (18.18%) with “3–5 co-habitants”]. The frequency of the need to break social isolation by at least one household member was not significantly higher in either EEG or HEG [530 participants (53.6%) vs. 1,644 participants (54.4%), respectively (*p* = 0.345)].

### Breaking social isolation

For the question “Do you consider that you are following the proper rules for social distancing?”, ~3,106 participants (77.57%) answered “yes,” with a slight difference between the number of participants who believed that they were not maintaining an adequate social distance in the EEG and HEG [273 participants (27.6%) and 625 participants (20.7%), respectively]. This difference was statistically different (*p* < 0.001). Regarding social distancing, 2,174 participants (54.2%) mentioned that they broke social isolation at least once per week in the last month, 1,306 participants (32.6%) stated that they went outside at least once in the last month, and only 524 participants (13.1%) did not go outside at all. Regarding how people who could not stay at home adapted their lives to follow social distancing measures as much as possible, 1,678 participants (41.9%) stated, “I go to work but remain outside home as less as possible” and 1,600 participants (39.9%) stated, “I leave home exclusively for essential activities.” The remaining participants stated, “I have decreased my activities involving exposure to a large number of people, but I keep going outside.”

After probing their behavior regarding social distancing, we asked the participants, “How many people in your closest circle do you consider not to be following adequate social distancing measures and behaving irresponsibly?”. All participants stated that they knew someone who was not following social distancing measures imposed by the government. A total of 2,257 participants (nearly 60%) stated that they knew >6 individuals who were not following social distancing measures. Only 172 participants (4.3%) stated that they had not interacted with people other than their co-habitants. Moreover, 3,831 participants (95.6%) mentioned interacting with >1 person who was not their co-habitant. Notably, 37% of participants interacted with >6 individuals and 20% interacted with >11 individuals.

After asking the participants about their degrees of social distancing and exposure, we asked them, “Based on your daily activities, what is the risk that you think you have of contracting COVID-19?” for which 2,690 participants (67.1%) answered “low-to-medium risk” and only 1,312 participants (32.7%) thought that they had a “high risk” of COVID-19 infection.

We also asked them why they could not adequately maintain social distancing measures and why they believed that the Mexican population could not maintain social distancing measures. These findings are presented in Table 2.

**Table 2:** Reasons why our sample broke social isolation rules.

	**Complete disinterest (*n* = 38)**	**Need to work (*n* = 2,050)**	**Despair and need for recreational activities (*n* = 453)**	**Family visits (*n* =702)**	**Errands (*n* =2,055)**	**I have not left home at all (*n* = 207)**
**Age groups**
18–25	18 (47.3%)	472 (23%)	300 (66.2%)	276 (39.3%)	663 (32.2%)	90 (71.5%)
26–50	17 (44.7%)	1,277 (62.2%)	132 (29.1%)	348 (49.5%)	936 (45.5%)	67 (32.3%)
50 or more	3 (7.8%)	301 (14.6%)	21 (4.6%)	78 (11.1%)	456 (22.1%)	50 (24.1%)
**Sex**
Male	17 (44.7%)	829 (40.4%)	228 (50.3%)	231 (32.9%)	596 (29%)	34 (16.4%)
Female	21 (55.2%)	1,241 (60.5%)	225 (49.6%)	471 (67%)	1,459 (70.9%)	173 (83.5%)
**School education**
Elementary	16 (42.1%)	520 (25.3%)	129 (28.4%)	169 (24.1%)	477 (23.2%)	55 (26.5%)
Higher	22 (57.8%)	1,530 (74.6%)	324 (71.5%)	533 (75.9%)	1,578 (76.7%)	152 (73.4%)

Participants were allowed to select at least one option and at most two options.

Preventive measures against the COVID-19 pandemic in Mexico: A cross-sectional study, México, 2020–2021.

### Personal preventive measures against COVID-19

We asked the participants how often they used preventive measures against COVID-19.

To our surprise, 3,852 participants (~96.20%) used preventive measures at least 50% of the time and 2,813 participants (almost 70.25%) stated that they used them all the time.

Approximately 70% of participants answered that more than half of the people they see on the street use preventive measures against COVID-19, and this included 1,087 participants who stated that < 25% of the people on the street use preventive measures.

We also asked them the reasons why they did not use preventive measures against COVID-19 all the time and their opinion on why they believed that Mexicans were not using preventive measures all the time. These findings are shown in Table 3. Table 4 presents the type of preventive measures against COVID-19 used by the study participants.

**Table 3:** Reasons why our sample did or did not use preventive measures against coronavirus.

	**Disinterest (*n* = 86)**	**It is tiring to use them (*n* = 418)**	**It is difficult for me to buy them (*n* = 221)**	**It is expensive to use them all the time (*n* = 181)**	**I do not think that you need to use them all the time (*n* =383)**	**I think it is unlikely that I will be infected by coronavirus (*n* = 112)**	**I use them all the time (*n* = 2,794)**
**Sex**
Male	32 (37.2%)	138 (33%)	104 (49.2%)	69 (38.1%)	167 (43.6%)	49 (43.7%)	871 (31.1%)
Female	54 (62.7%)	280 (66.9%)	117 (55.4%)	112 (61.8%)	216 (56.3%)	63 (56.2%)	1,923 (68.8%)
**School education**
Elementary	61 (70.9%)	138 (138%)	75 (35.5%)	74 (40.8%)	126 (32.8%)	34 (30.3%)	575 (20.5%)
Higher	25 (29.1%)	280 (66.9%)	146 (69.1%)	107 (59.1%)	257 (67.1%)	78 (69.6%)	2,219 (79.4%)
**Occupation**
Formal job	38 (44.1%)	231 (55.2%)	146 (69.1%)	109 (60.2%)	246 (64.2%)	48 (42.8%)	1,652 (59.1%)
Informal job	16 (18.6%)	36 (8.6%)	29 (13.7%)	31 (17.1%)	40 (10.4%)	13 (11.6%)	175 (6.2%)
Student	21 (24.4%)	107 (25.5%)	29 (13.7%)	28 (15.4%)	74 (19.3%)	31 (27.6%)	581 (20.7%)
Retired	3 (3.4%)	11 (2.6%)	1 (0.4%)	2 (1.1%)	13 (3.3%)	6 (5.3%)	101 (3.6%)
Neither working nor studying	6 (6.9%)	13 (3.1%)	8 (3.6%)	6 (3.3%)	0	6 (5.3%)	107 (3.8%)
Other	2 (2.3%)	20 (4.7%)	8 (3.6%)	5 (2.7%)	7 (6.2%)	8 (7.1%)	178 (6.3%)

Participants were allowed to select at least one option and at most two options.

Preventive measures against the COVID-19 pandemic in Mexico: A cross-sectional study, México, 2020–2021.

**Table 4:** Preventive measures usage against COVID-19 by educational level.

	**Elemental education group (*n* = 987)**	**Higher education group (*n* = 3,017)**	**Total**
**Face masks**
Certified face mask	495 (50.1%)	1,563 (51.8%)	2,058 (51.9%)
Non-certified face mask	482 (48.8%)	1,444 (47.8%)	1,926 (48.1%)
I do not use any face mask	10 (1%)	10 (0.3%)	20 (49.9%)
**Mask/safety eyewear**
Certified face mask or safety eyewear	270 (27.3%)	746 (24.7%)	1,016 (23.3%)
Non-certified safety eyewear	225 (22.7%)	853 (28.2%)	1,078 (26.9%)
I do not use any safety eyewear	492 (49.8%)	1,418 (47%)	1,910 (47.7%)
**Alcohol-based sanitizer**
Certified alcohol-based sanitizer	820 (83%)	2,582 (85.5%)	3,402 (84.9%)
Non-certified alcohol-based sanitizer	132 (13.3%)	399 (13.2%)	531 (13.2%)
I do not use any sanitizer	35 (3.5%)	36 (1.1%)	71 (1.7%)

Certified face masks are defined as those distributed by pharmaceutical companies, pharmacies, or healthcare personnel. Non-certified face masks defined as those custom-made, homemade, or non-certified for healthcare personnel use.

Preventive measures against the COVID-19 pandemic in Mexico: A cross-sectional study, México, 2020–2021.

### COVID-19 screening test

Considering the Mexican population's exposure to COVID-19, it is essential to know how many participants in this study had been tested for COVID-19 to determine if they were following a good preventive healthcare program. A total of 3,502 participants (~87.46%) had never undergone COVID-19 testing, and of the remaining 502 participants (12.53%), 389 had a negative test result and only 133 had a positive test result. When we asked the participants, “Do you know anybody who has presented symptoms of or has been diagnosed with COVID-19?”, 2,880 participants (71.92%) reported knowing at least one person who had been diagnosed with COVID-19.

### The Mexican healthcare system

We asked the participants if they believed that hospitals in Mexico were prepared to face the pandemic crisis, pointing out the differences between the public and private healthcare systems. A total of 2,501 (62.46%) and 2,676 participants (66.83%) believed that public and private hospitals were prepared for the pandemic, respectively. By level of education, 1,919 participants (63.60%) from the HEG and 582 participants (58.9%) from the EEG responded that they believed that the public healthcare system was prepared for the pandemic (*p* < 0.01).

Furthermore, 2,043 participants (67.71%) of the HEG and 633 participants (64.13%) of the EEG responded that they believed that private hospitals were prepared for the pandemic (*p* < 0.05).

Then, we asked the participants about the factors they thought influenced the Mexican healthcare system's inability to be prepared for a pandemic. The participants could select more than one answer for this question. The most selected answer (65.58% of participants) was “The facilities and equipment required to deal with the pandemic situation are not available” followed by “The health-care personnel do not have enough supplies” (59.94% of participants) and “There are not enough health-care personnel to serve the entire population” (43.32% of participants). Only 5.49% of participants thought that the doctors and healthcare personnel were not sufficiently prepared to face the pandemic.

## Discussion

### The general state of Mexico and its population

When attempting to comprehend the spread of COVID-19 in Mexico, we must consider the baseline characteristics of our population. Approximately 12.5% of the country's population, or ~15.4 million people, were aged 60 or older, and at least 69.4% had some form of disability or comorbidity, according to demographic studies (11). By August 2020, of the almost 57,000 registered deaths due to COVID-19 in Mexico, 58% of cases were adults aged >60 years (12). In China, the overall case fatality rate reported as of February 2020 was 8% among patients aged 70–79 years and 14.8% among those aged >80 years (13). Therefore, older adults are advised to remain at home and maintain social isolation to prevent COVID-19 infection. In our study, 729 participants (~18.2%) were in this susceptible age group.

Another factor that makes the Mexican population highly vulnerable to COVID-19 infection is the high incidence of chronic diseases and metabolic disorders. This is represented in our study, as 1,803 participants (43%) had at least one comorbidity. The available data associate baseline comorbidities with a severe course of COVID-19 (14) and interrupting anti-hypertensive treatment can precipitate cardiovascular decompensation (15). It has been studied that the SARS-CoV-2 virus uses angiotensin-converting enzyme 2 (ACE2) receptors on the lungs to cause infection, directly infecting other cells by regulating blood pressure. As hypertension patients may exhibit alterations in the structure or expression of ACE2 receptors, this could facilitate a more severe infection (16). In another Mexican study, of ~3,73,963 adults with COVID-19, 16.1% had diabetes, the predicted probability of hospitalization was 38.4% for patients with diabetes only and 42.9% for those with diabetes and one or more comorbidities (17). The comorbidities that most increased the risk of intensive care unit stay and intubation were diabetes, immunosuppression, and obesity (18).

Regarding the prevalence of obesity and overweight, a high prevalence is observed for the population aged 12–19 years (17%) and >20 years (42%) in the northern region of Mexico (19). In this study, 1,272 participants (31.7%) had a diagnosis of obesity. A previous study showed that obesity is a risk factor for hospitalization, admission to the intensive care unit, and the development of severe consequences that lead to death in cases of COVID-19 (20). A French study showed that severely obese patients (body mass index >35) require invasive mechanical ventilation more frequently than lean patients, regardless of age, sex, diabetes, and high blood pressure (21). Considering the Mexican population's health condition, it is easy to understand why the COVID-19 fatality rate reached such high levels and why the prevention policies were directed toward isolating the population at risk, representing a large percentage of the Mexican population.

Nevertheless, probably the most critical factors to influence the COVID-19 pandemic's development in Mexico are the status of the healthcare system and government strategies. Accordingly, a study published in December 2020 strongly criticized Mexico's position on the pandemic. It stated that Mexico's vast inequality, underfunded healthcare system, sizeable informal economy, and multigenerational housing made it particularly vulnerable to the spread of the virus. However, a lack of strategy, combined with the president's mixed messages, has exacerbated the situation in a poorly equipped country to handle a pandemic (22). Regarding this lack of a sound healthcare system, we asked the participants about their confidence in the Mexican healthcare system; almost 40% of the participants disapproved of the system. This finding indicated that almost half of our study participants considered the Mexican healthcare system as inferior and believed that a lack of support to the institutions to enable doctors, who were already overwhelmed in every way, to perform their work was a problem. Moreover, because of these issues, it was reported that Mexico was the number one country in the mortality of health personnel related to COVID-19 (23, 24). The issues discussed in this section collectively represent the general conditions that have led to high COVID-19 infection and death rates in Mexico.

### The attitude of the Mexican population toward the pandemic and their preventive measures

Undoubtedly, the factors that maintain the virus spreading in developing countries are overpopulation, overcrowding, and insufficient health services. In our study, over 75% of the participants lived with >3 co-habitants, of which at least 87.4% had one household member who frequently broke social isolation. Even when the participants were constantly exposed to not quarantined people, almost three out of four stated that they were maintaining adequate social distancing measures. However, almost 60% of participants stated that they knew at least six individuals who were not following proper quarantine measures. Regarding the effectiveness of being quarantined, a study conducted in China concluded that latent individuals' contact rate is between 6 and 18 persons, representing the possible impact of isolation and quarantine measures on the disease infection rate. These findings suggest that interventions, such as isolation and quarantine, can effectively reduce the peak number of COVID-19 infection cases and delay infection cases' peak time by reducing the contact rate (25). Another study showed that the infection's epidemic trend mainly depends on quarantine and suspected cases (26). Worldwide, it appeared easier to stay in social isolation during the early stage of COVID-19 spread.

As the year 2020 progressed, however, maintaining strict isolation and avoiding large gatherings and events became more difficult, and people were less inclined to adhere to social distancing measures. People no longer desired to comply with government directives to maintain social distance due to the negative effects of social distancing as a result of lifestyle changes brought about by the quarantine, such as decreased physical activity and unhealthy eating habits (27). Our study participants could not perform strict social distancing measures, presented in Table 2, and include the need to keep their jobs, maintain their daily routine, and help family members.

In a country where general conditions make a prolonged quarantine unrealistic, the use of preventive measures against the virus becomes a mainstay to control its spread. When asked how often they used COVID-19 prevention measures, most of our study participants said frequently. However, 70% of participants said < 50% of co-habitants used such measures. Some reports on global behavior regarding the use of face masks stated that Mexico is one of the countries with the maximum use of masks (28, 29). The Mexican government confirmed this (29) and it is in agreement with our findings that ~99.5% of the studied population used face masks, however, only 50% used a certified mask and the remainder used a homemade or handmade mask that did not meet quality standards and did not confer or guarantee any protection to the user or people around. This means that only one in two Mexicans uses the WHO's primary protection tool against the coronavirus (30). The use of face masks is supported by science because coronavirus transmission is mainly through aerosol drops exhaled from infected patients, whether symptomatic or not. A study regarding face mask use concluded that it is reasonable to suggest that face masks can mitigate the current pandemic because they may reduce coronavirus particles in aerosols and respiratory droplets (31). That is why using measures that protect from aerosols and droplets is also important, such as protective glasses and other equipment. However, the low degree of acceptance of eye protection in our population was alarming. The reasons why our study population did not use the different types of preventive measures against COVID-19 are presented in Tables 3, 4, and they indicate an economic, social, and educational reality that must be fought with information and health education; otherwise, it will be impossible to stop the COVID-19 contagion.

The WHO has strongly emphasized the importance of carrying out tests to detect cases timely, predominantly asymptomatic cases, and isolate them (31). However, in developing countries such as Mexico, screening campaigns are limited, and thus it is decided to opt for different models. For example, a model designed at the University of Guadalajara, Mexico, has been used in Jalisco, Mexico, and it detects 100% of suspected cases with mild symptoms, unlike the Sentinel model proposed by the federal government, which randomly samples 1 out of 10 suspected patients and does not have a specific screening method in COVID-19 concentrated areas (32). When comparing the number of COVID-19 tests made by country among Latin American countries performed in January 2021, Mexico was ranked 7th, whereas Brazil, Peru, and Colombia were the top-ranked countries. In a world ranking evaluated in December 2020 of tests per million inhabitants, Mexico was ranked 24, in a ranking led by the United Kingdom, the United States, and Russia. A total of 3,502 participants (~87.46%) in our study had never undergone COVID-19 testing, but 2,880 participants (71.92%) reported knowing at least one person who had been diagnosed with COVID-19 (33).

The COVID-19 pandemic situation that Mexico faces is a sum of multiple negative factors enhanced by an unexpected event. Moreover, it is impossible to point out a single challenge to overcome because, even though management at different levels has left things to be desired, the general conditions of the population and the Mexican healthcare system's state could not have been repaired rapidly even with different management policies (34). It is necessary to attack the problems at their roots by starting health education campaigns for the population that will enable people to identify the risk to which they are exposed, help them decide to be part of the strategy to stop the pandemic, and achieve general habits that can help improve the incidence of chronic diseases and obesity (35, 36). It is also essential to improve the medical staff's conditions and provide a better hospital infrastructure. Mexico's leaders must reflect on their stance against the coronavirus and look for ways to increase the provision of aid exponentially. We believe that for Mexico to overcome the current pandemic situation, everyone must be involved to stop the rapid spread of the virus. Tools must be provided to people so that the preventive measures they decide to use are realistic, such as providing monetary support so that people can quarantine without the need to go out to earn their livelihood (37).

The work presented has certain limitations. Even though we compiled data from a large sample, it is possible that our findings are not representative of the entire country's population. Our study design can only generate the hypothesis that the decision of the Mexican population not to take adequate preventative measures, to break isolation, and to conduct inadequate testing could be a significant factor in the disease's spread and damage.

## Conclusion

The COVID-19 pandemic outcomes in Mexico are the result of multiple negative factors, such as high rates of comorbidities, a high number of people living together at home, many people breaking social isolation, and most of the population using non-certified preventive measures, which may not have the necessary effectiveness. We also found inadequate epidemiological monitoring with evidence in the case of our study population. Taken together, this indicates a complicated COVID-19 situation for Mexico.

## Data availability statement

The raw data supporting the conclusions of this article will be made available by the authors, without undue reservation.

## Ethics statement

The authors assert that all procedures contributing to this work comply with the relevant national and institutional committees' ethical standards on human experimentation and with the Helsinki Declaration of 1975, as revised in Fortaleza, Brazil 2013. This study protocol was reviewed and approved by the Local Committee in Ethics and Research with the approval number 2022-1301-140. Also, the study protocol was submitted to ClinicalTrials.gov and registered with the identifier: NCT04771117. The surveys were anonymous to guarantee the confidentiality. Written informed consent was obtained from each participant.

## Author contributions

JI-P and AG-O conceived the study. TC-V, AC-V, JC-C, ER-E, OR-L, RZ-O, EC-P, and JS-F prepared the study material. ML-Z, RM-A, CF-O, YA-M, JG-B, and PF-B assisted with the student recruitment process and distribution of the surveys. IO-R, RN-H, FB-C, JI-P, and AG-O performed data management and analysis. JI-P, GC-G, CF-O, and AG-O wrote the first draft of the manuscript. All authors contributed to the study's conception and design, critiqued all versions of the manuscript, read, and approved the final manuscript.

## Conflict of interest

The authors declare that the research was conducted in the absence of any commercial or financial relationships that could be construed as a potential conflict of interest. The handling editor FG-S declared a shared affiliation with the authors FB-C, JI-P, YA-M, JC-C, IO-R, RN-H, TC-V, AC-V, ER-E,RZ-O, JS-F, JG-B, PF-B, CF-O, and AG-O at the time of review.

## Publisher's note

All claims expressed in this article are solely those of the authors and do not necessarily represent those of their affiliated organizations, or those of the publisher, the editors and the reviewers. Any product that may be evaluated in this article, or claim that may be made by its manufacturer, is not guaranteed or endorsed by the publisher.

## Abbreviations

ACE2: angiotensin-converting enzyme 2
COVID-19: coronavirus disease 2019
EEG: elementary education group
FCV-19S: fear of COVID-19 scale
HEG: higher education group
WHO: World Health Organization.
