# Supplementary material for: Preventive measures against the COVID-19 pandemic in Mexico: A cross-sectional study
Source: Front Public Health. 2022 Oct 11;10:932010. doi: 10.3389/fpubh.2022.932010 (PMC9596139; doi:10.3389/fpubh.2022.932010)
Supplement: Supplementary file 1 [file Data_Sheet_1.pdf]

Supplementary information: Additional file 1
Title: Preventive measures against the COVID-19 pandemic in Mexico survey.
Description: Survey applied to identify the risk factors favoring the COVID-19 contagion by studying the Mexican population's quarantine behavior and preventive measures.
Sex: Male/Female
Age:
State of residence:
Occupation:
a. Formal job
b. Informal job
c. Retired
d. Student
e. Neither working nor studying
f. Other

Education level:
a. Elementary school
b. High school
c. Middle school
d. College
e. Master's degree

Do you have any health condition?
None/Overweight/Obesity/Diabetes/Hypertension/Chronic kidney disease/Chronic obstructive pulmonary disease (COPD)/Posttransplantion/Cardiovascular disease/Human immunodeficiency virus infection/Other

Do you consume any of the following substances?
a. Tobacco
b. Alcohol
c. Cannabis or other drugs
d. Coffee
e. None

How many people live in your household?
a. 1–2
b. 3–5
c. 6 or more

How many of the people living in your household frequently find themselves in need of breaking social isolation and leaving home?
a. None
b. 1–2 people
c. 3–5 people
d. 6 or more people

With how many people do you interact with daily?
a. None
b. 1–5 people
c. 6–10 people
d. 11 or more

Based on your daily activities, what is the risk that you think you have of contracting COVID-19?

a. Extremely high
b. High
c. Regular
d. Low
e. None

How have you been practicing social distancing since the quarantine was established in Mexico?

a. I have continued my life in a normal way
b. I have decreased my activities involving exposure to many people, but I keep going outside
c. I go to work but remain outside home as less as possible
d. I leave home exclusively for essential activities (e.g., getting groceries to continue social isolation)
e. I do not leave home at all

How often do you break social isolation and go out for an activity in which you interact with more people?

a. I have not broken social isolation
b. Less than once a week, but more than once in a month
c. Once per week
d. Two or three times a week
e. More than four times a week

Why do you break social isolation? **Select the more likely option according to you, maximum two answers.**

a. Disinterest
b. Need to work
c. Despair and need for recreational activities
d. Family visits
e. Errands
f. I have not left home at all

What do you think leads the Mexican population to break social isolation? **Select the more likely option according to you, maximum two answers.**

a. Disinterest
b. Need to work
c. Despair and need for recreational activities
d. Family visits
e. Errands

How many people in your closest circle do you consider not to be following adequate social distancing measures and behaving irresponsibly?

a. 1–2 people
b. 3–5 people
c. 6 or more

Do you consider that you are following the proper rules for social distancing?
a. Yes
b. No

Do you use preventive measures against coronavirus daily (e.g., alcohol-based hand sanitizer, face masks, face shields, and safety eyewear)?
a. Always (all the time)
b. Almost always (75% of the time)
c. Sometimes (50% of the time)
d. Almost never (25% of the time)
e. Never (0% of the time)
f. I never leave home

Why do you not use preventive measures against coronavirus all the time? **Select the more likely option according to you, maximum two answers.**
a. Disinterest
b. It is tiring to use them
c. It is difficult for me to buy them
d. It is expensive to use them all the time
e. I do not think that you need to use them all the time
f. I think it is unlikely that I will be infected by coronavirus
g. It does not apply: I use them all the time

What percentage of the people that you see in the streets, at work or office, etc., use preventive measures against coronavirus (e.g., alcohol-based hand sanitizer, face masks, face shields, and safety eyewear)?
a. All of them (100% of the people)
b. Majority (approximately 75% of the people)
c. Half the people (approximately 50% of the people)
d. Few people (approximately 25% of the people)
e. Almost nobody (approximately <10% of the people)

Why do you think that the Mexican population does not use coronavirus preventive measures all the time? **Select the more likely option according to you, maximum two answers.**
a. Disinterest
b. It is tiring to use them
c. It is difficult for the population to buy them
d. It is expensive for the population to use them all the time
e. People do not think that they need to use the measures all the time
f. People do not think that they are likely to be infected by coronavirus

Depending on the category, select the type of protection method that you use the most:
Face masks
a. Certified face mask (e.g., Triple layer mask, N95, KN95)
b. Noncertified face mask (e.g., fiber mask, carbon fiber face mask, handcrafted face mask, scarfs, painted mask)
c. I do not use any face mask

Mask/safety eyewear

a. Certified mask or safety eyewear (e.g., distributed by pharmaceutical companies, pharmacies, or health-care personnel)
b. Noncertified mask or safety eyewear (e.g., acetate face shield, homemade face mask, or handcrafted face mask)
c. I do not use any mask/safety eyewear

Alcohol-based sanitizer

a. Certified alcohol-based sanitizer (e.g., distributed by pharmaceutical companies, pharmacies, or health-care personnel)
b. Noncertified alcohol-based sanitizer (e.g., homemade sanitizer, custom made sanitizer)
c. I do not use any alcohol-based sanitizer

Do you stay informed about the pandemic in your country and state?

a. Yes, I stay informed
b. Just a bit, I try to stay informed
c. I prefer to avoid the news because it makes me feel anxious
d. I am not interested

Do you know what are the symptoms of COVID-19?
Yes/No

Do you know anybody who has presented symptoms of or was diagnosed with COVID-19?
Yes/No

Have you been tested for COVID-19?
Yes/No

If you have been tested, what was the result?
Positive/Negative/I have not been tested.

Do you know where to go in case of presenting COVID-19 symptoms?
Yes/No

Do you consider that health-care personnel of public hospitals are well trained to treat patients with COVID-19?
Yes/No

Which of the following do you consider as the most important factor for public hospitals to deal with the pandemic? **Select the more likely option according to you, maximum two answers.**

a. Health-care personnel are not well prepared
b. Health-care personnel do not have enough supplies
c. There are not enough health-care personnel to serve the entire population
d. The facilities and equipment required to deal with the pandemic situation are not available

Do you consider that health-care personnel of private hospitals are well trained to treat patients with COVID-19?
Yes/No
